# Cohort profile: Copenhagen Hospital Biobank-chronic inflammatory disease—inflammatory bowel disease (CHB-CID:IBD) genetic cohort

**DOI:** 10.1007/s10654-025-01239-4

**Published:** 2025-06-09

**Authors:** Behrooz Darbani, Thorsten Brodersen, Anette Liljensøe, Signe Bek Sørensen, Josephine Bjergbæk Olsson-Svendsen, Alfonso Buil, Ahmed Kamal, Andrew J. Schork, Anja Poulsen, Bertram Dalskov Kjerulff, Bitten Aagaard, Britta Ørnfelt Lund, Charlotte Siggaard Rittig, Christina Mikkelsen, Daniel Millencourt Larsen, David Westergaard, Ditte Rudbeck-Resdal, Dorte Helenius Mikkelsen, Else Randers, Frank Vinholt Schiødt, Hans Jürgen Hoffmann, Isabella Friis Jørgensen, Ivan Brandslund, Jacob Broder Brodersen, Jakob Hjorth von Stemann, Jakob Thaning Bay, Janna Nissen, Jeanette Sørensen, Jens Kjærgaard Boldsen, Joseph Dowsett, Josephine Gladov, Karina Banasik, Kathrine Agergård Kaspersen, Katrine Carlsen, Khoa Manh Dinh, Lauge Kellermann, Lea Arregui Nordahl Christoffersen, Liam James Elgaard Quinn, Lise Wegner Thørner, Lone Larsen, Luise Aamann, Malene Rohr Andersen, Maria Didriksen, Maria Joanna Alexandraki, Marianne Kragh Thomsen, Mette Julsgaard, Mette Nyegaard, Michael Schwinn, Mie Topholm-Bruun, Mikael Njai Leite, Morten Lee Halling, Natalia Pedersen, Ole K. Bonderup, Palle Duun Rohde, Pernille Dige Ovesen, Ram Benny Dessau, Sanaz Saboori, Sofie Holm-Christensen, Steffen Bank, Susan Mikkelsen, Thomas Folkmann Hansen, Thomas Werge, Niels Qvist, Erik Sørensen, Johan Burisch, Merete Lund Hetland, Bente Glintborg, Christian Erikstrup, Søren Brunak, Henrik Ullum, Sisse Rye Ostrowski, Ole Birger Vesterager Pedersen, Vibeke Andersen

**Affiliations:** 1 Molecular Diagnostics and Clinical Research Unit, Department of Internal Medicine, University Hospital of Southern Denmark, Aabenraa, Denmark; 2 Department of Clinical Immunology, Zealand University Hospital, Køge, Denmark; 3 Mental Health Centre, Sct. Hans, Institute of Biological Psychiatry, Copenhagen University Hospital, Roskilde, Denmark; 4 Department of Internal Medicine, Zealand University Hospital, Køge, Denmark; 5 Mental Health Services, Institute of Biological Psychiatry, Copenhagen University Hospital, Roskilde, Denmark; 6 Department of Hepatology and Gastroenterology, Zeeland University Hospital, Køge, Denmark; 7 Department of Clinical Immunology, Aarhus University Hospital, Aarhus, Denmark; 8 Department of Clinical Immunology, Aalborg University Hospital, Aalborg, Denmark; 9 Emergency Medicin, Esbjerg Hospital, Esbjerg, Denmark; 10 Department of Paediatrics and Adolescent Medicine, Aarhus University Hospital, Aarhus, Denmark; 11 Department of Clinical Immunology, Copenhagen University Hospital – Rigshospitalet, Copenhagen, Denmark; 12 Faculty of Health and Medical Science, Novo Nordisk Foundation Center for Basic Metabolic Research, University of Copenhagen, Copenhagen, Denmark; 13 Emergency Medicine, Nord Zealand Hospital, Hillerød, Denmark; 14 Department of Obstetrics and Gynaecology, Copenhagen University Hospital – Amager and Hvidovre Hospital, Hvidovre, Denmark; 15 Department of Hepatology and Gastroenterology, Aarhus University Hospital, Aarhus, Denmark; 16 Department of Internal Medicine, Viborg Regional Hospital, Viborg, Denmark; 17 Digestive Disease Center, Bispebjerg Hospital, Cophenhagen, Denmark; 18 Department of Clinical Medicine, Aarhus University, Aarhus, Denmark; 19 Faculty of Health and Medical Sciences, Novo Nordisk Foundation Center for Protein Research, University of Copenhagen, Copenhagen, Denmark; 20 Department of Biochemistry and Immunology, University Hospital of Southern Denmark, Odense, Denmark; 21 Department of Regional Health Research, University of Southern Denmark, Esbjerg, Denmark; 22 Department of Internal Medicine, Section of Gastroenterology, Esbjerg Hospital – University Hospital of Southern Denmark, Esbjerg, Denmark; 23 Department of Gastroenterology and Hepatology, Aalborg University Hospital, Aalborg, Denmark; 24 Department of Clinical Biochemistry, Herlev and Gentofte Hospital, Hellerup, Denmark; 25 BERTHA - the Danish Big Data Centre for Environment and Health, Aarhus University, Aarhus, Denmark; 26 Department of Paediatrics and Adolescent Medicine, Copenhagen University Hospital – Herlev and Gentofte Hospital, Copenhagen, Denmark; 27 Department of Gastroenterology and Hepatology, Herlev Hospital – University of Copenhagen, Herlev, Denmark; 28 Department of Clinical Medicine, Center for Molecular Prediction of Inflammatory Bowel Disease (PREDICT), Aalborg University, Aalborg, Denmark; 29 Department of Gastroenterology and Hepatology, Goedstrup Hospital, Herning, Denmark; 30 Department of Clinical Microbiology, Aarhus University Hospital, Aarhus, Denmark; 31 Department of Health Science and Technology, Faculty of Medicine, Aalborg University, Aalborg, Denmark; 32 Clinical Immunology Research Unit, Department of Clinical Immunology, Odense University Hospital, Odense, Denmark; 33 The Department of ORL – Head and Neck Surgery, Odense University Hospital, Odense, Denmark; 34 Department of Internal Medicine, Section of Gastroenterology, Esbjerg and Grindsted Hospital, Esbjerg, Denmark; 35 Department of Gastroenterology, Slagelse Hospital, Slagelse, Denmark; 36 Diagnostisc Center, Silkeborg Hospital, Silkeborg, Denmark; 37 Department of Clinical Microbiology, Zealand University Hospital, Køge, Denmark; 38 Institute of Regional Health Research, University of Southern Denmark, Odense, Denmark; 39 Present Address: Department of Emergency, Aarhus University Hospital, Aarhus, Denmark; 40 Regional Pharmacy, Aarhus University Hospital, Aarhus, Denmark; 41 Neurogenomic Group, Translational Research Centre, Copenhagen University Hospital – Rigshospitalet, Glostrup, Denmark; 42 Department of Clinical Medicine, Faculty of Health and Medical Sciences, University of Copenhagen, Copenhagen, Denmark; 43 Reserach Unit for Surgery, Odense University Hospital, Odense, Denmark; 44 Gastro Unit, Medical Section, Copenhagen University Hospital – Amager and Hvidovre, Hvidovre, Denmark; 45 Copenhagen Center for Inflammatory Bowel Disease in Children, Adolescents and Adults, Copenhagen University Hospital – Amager and Hvidovre, Hvidovre, Denmark; 46 The DANBIO Registry and the Danish Rheumatologic Biobank, Center for Rheumatology and Spine Diseases, Centre for Head and Orthopaedics, Rigshospitalet, Glostrup, Denmark; 47 Copenhagen Center for Arthritis Research (COPECARE), Center for Rheumatology and Spine Diseases, Centre of Head and Orthopaedics, Rigshospitalet, Glostrup, Denmark; 48 Statens Serum Institut, Copenhagen, Denmark; 49 Institute of Molecular Medicine, University of Southern Denmark, Odense, Denmark; 50 University of Southern Denmark, Odense, Denmark; 51 Present Address: Global Medical Affairs, ALK-Abelló A/S, Hørsholm, Denmark

**Keywords:** Crohn’s disease, Faecal Calprotectin, Genetic cohort, Genetic relatedness, Inflammatory bowel disease, Ulcerative colitis

## Abstract

The Copenhagen Hospital Biobank-chronic inflammatory disease—inflammatory bowel disease (CHB-CID: IBD) cohort contributes to genetic research in inflammatory bowel disease, including Crohn’s disease and ulcerative colitis. Of the 327,084 enrolled and genotyped individuals in the cohort, 10,626 have been diagnosed with IBD as of May 2023. The CHB-CID: IBD cohort includes both patients without IBD and healthy blood donors as control groups. Clinical data is collected from Danish registries and patient records, including details on hospital contacts, co-morbidities, medication, surgical procedures, and laboratory investigations. The cohort features a wide age range (> 18 years), extensive population coverage representative of Danish adults, and validated IBD diagnoses. Finally, the cohort benefits from continuous recruitment and regular updates of clinical information. The aim is to enhance IBD management and ultimately improve patients’ quality of life.

## Background and rationale

Genetic and non-genetic factors can contribute to the development and progression of diseases. The multifactorial nature of disease development is well-known in chronic inflammatory diseases and leads to clinical heterogeneity among patients. Therefore, understanding disease mechanisms and developing efficient treatments and preventive solutions is challenging when relying solely on small-scale studies. With a strategic outlook to disease prevention, we describe a cohort for inflammatory bowel disease [IBD; Crohn’s disease (CD) and ulcerative colitis (UC)]. IBDs are chronic gastrointestinal tract disorders with increasing incidence [1] and recent prevalence of 0.3% to 0.8% varying among populations [2, 3].

Higher risks have been reported for close relatives of patients with IBD [4] and more than 200 genetic variants identified as being associated with IBD [5–8]. Along with genetic risks, environmental factors such as processed and protein-based foods, smoking, and gut microbes also play a role in IBD pathogenesis [8–11]. These factors and the unsatisfactory responses to current treatments, treatment side-effects, and substantial socioeconomic burden to the health care system and patients [12, 13] call for large-scale studies to better understand IBD development, progression and clinical heterogeneity and to develop new diagnostic classifications and personalised treatments. The overall aim of the CHB-CID:IBD cohort is to enhance the management of IBD by generating research outcomes that can be translated into clinical tools. We will integrate genetic and clinical data to redefine and reclassify IBD phenotypes based on specific pathophysiological mechanisms. Based on this approach, we will provide models to improve diagnosis and to predict disease risk, disease outcomes, comorbidities, drug adverse effects, and treatment responses.

## Study design: population, recruitment, and data collection

**Fig. 1:**
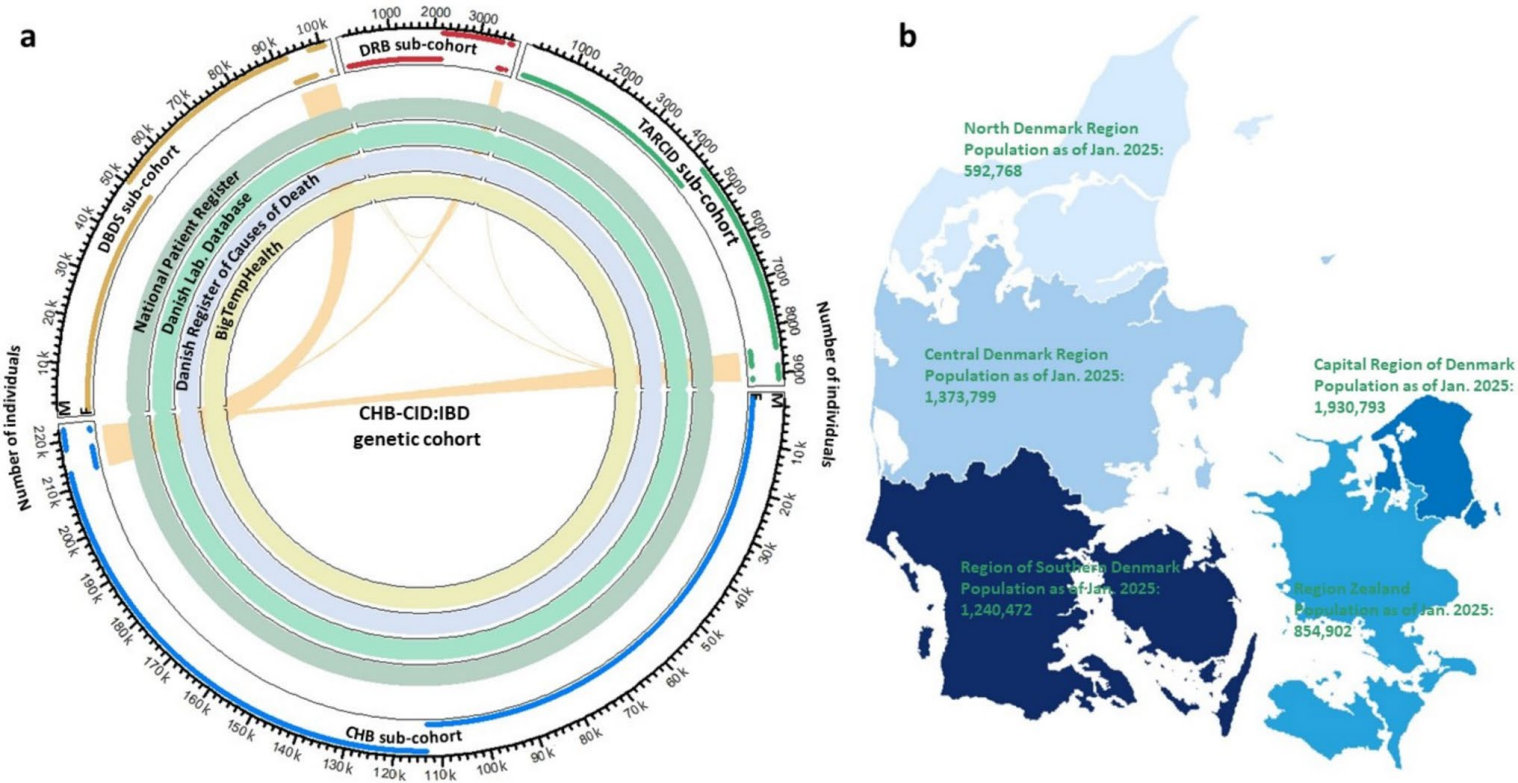
CHB-CID:IBD genetic cohort structure and its linked clinical databases from all five healthcare regions of Denmark. **a** The links among sub-cohorts represent individuals recruited to multiple sub-cohorts. F: Females (bottom line), M: Males (upper line), CHB: Copenhagen Hospital Biobank, DBDS: Danish Blood Donor Study, the DRB: Danish Rheumatologic Biobank (The Biomarker Protocol), TARCID: Targeted Treatment of Chronic Inflammatory Disease. **b** A map of Denmark healthcare regions, adapted from Danmarks Statistik (https://www.dst.dk/)

The CHB-CID:IBD is a large cohort of IBD cases and non-IBD controls contributing clinical and genetic data for developing molecular diagnostic approaches and effective treatment solutions to IBDs. All CHB-CID:IBD participants are genotyped at the time of study entry. The cohort utilises information from four sub-cohorts (see the sub-cohorts below), the Danish National Health Registries [14], and data extracted from electronic health records in BigTempHealth [15] (Fig. 1a). The information from Danish registries includes age, gender, hospital diagnoses, laboratory tests, procedures, and surgeries, described in detail in the 'Clinical and laboratory measurements' section below. The current registry data reflects the most recent update as of May 2023.

The cohort is composed of four sub-cohorts: the Copenhagen Hospital Biobank (CHB), the Danish Blood Donor Study (DBDS), the Danish Rheumatologic Biobank (DRB), and Targeted Treatment of Chronic Inflammatory Disease (TARCID) as shown in Fig. 1a. The CHB sub-cohort [16] is based on the CHB study “Developing the Basis for Personalized Medicine in Chronic Inflammatory Diseases (CHB-CID)”. CHB was initiated in February 2009 and is based on collecting leftover material from routine blood analyses in hospitals of the Capital Region of Denmark (see Fig. 1b). The CHB sub-cohort includes individuals diagnosed with IBDs (n = 6030: Females = 3333 & Males = 2697, see Table 1). The DBDS sub-cohort [17, 18] was initiated in March 2010 and includes blood donors recruited at Danish blood banks after giving informed consent. Blood donors are healthier than the general population, manifesting epidemiologically the healthy donor effect [17]. Still, over time, 585 donors have been diagnosed with incident IBD (Females = 284 & Males = 301, see Table 1). The Danish Rheumatological Biobank (DRB) sub-cohort [19, 20] was established in May 2015 and includes 149 individuals with IBDs (Females = 78 & Males = 71, see Table 1). The TARCID sub-cohort started in 2009 and includes patients with chronic inflammatory disease, most of whom have been treated with Tumor Necrosis Factor Inhibitors (TNFi) (IBD: n = 4214: Females = 2228 & Males = 1986, see Table 1). Clinical data was extracted from the patient records and includes information on outcomes from TNFi as described previously [21–23].

**Table 1:** CHB-CID:IBD cohort statistics

	IBDs and non-IBDs		non-IBD controls		IBDs		UCs	CDs
	Ind.^c^	Age^d^; F%^e^	Ind.^c^	Age^d^; F%^e^	Ind.^c^	Age^d^; F%^e^	Ind.^c^; F%^e^	Ind.^c^; F%^e^
CHB	222,390	67.0; 53.2	214,413	67.2; 53.1	6030	59.3; 55.3	3743; 54.6	2287; 56.3
CHB-only^a^	213,163	67.6; 53.0	205,657	67.9; 52.9	5611	59.9; 55.4	3509; 54.7	2102; 56.5
DBDS	101,068	49.5; 49.8	100,196	49.5; 49.8	585	48.1; 48.5	403; 49.1	182; 47.3
DBDS-only^a^	92,617	49.4; 49.1	91,873	49.4; 49.0	495	48.5; 49.9	345; 49.3	150; 51.3
DRB	3732	64.2; 62.5	3545	64.6; 62.9	149	55.4; 52.3	81; 50.6	68; 54.4
DRB-only^a^	3524	64.6; 62.4	3357	64.5; 62.8	130	55.4; 53.1	71; 50.7	59; 55.9
TARCID	9153	56.3; 55.1	4666	61.2; 57.1	4214	50.7; 52.8	1962; 51.0	2252; 54.4
TARCID_only^a^	8499	56.3; 54.9	4367	61.2; 57.0	3952	50.5; 52.5	1825; 50.7	2127; 54.2
All (CHB-CID:IBD)^b^	327,084	61.8; 52.1	314,224	61.9; 52.1	10,626	55.6; 54.0	5994; 53.0	4632; 55.2

^a^Statistics on non-overlapped individuals (not recruited into the other three sub-cohorts). ^b^The difference in the number of individuals between [IBDs and non-IBDs] and ([non-IBD controls] + [IBDs]) accounts for the filtered individuals based on IBD definition, i.e., individuals with less than two IBD diagnoses or with IBD diagnoses except type A and B. ^c^Number of individuals. ^d^Average age in years (for alive individuals as of May 2023 and for deceased individuals as of death date). ^e^F%: (Females/ (Males + Females)) × 100. IBD: inflammatory bowel disease, CD: Crohn’s disease, UC: Ulcerative colitis, and Ind.: Individuals

IBD cases were identified and classified using information from National Patient Register and based on the International Statistical Classification of Diseases and Related Health Problems revision 8 (ICD8) for diagnoses between 1969 and 1993 and version 10 (ICD10) for diagnoses from 1994 onwards. We applied a previously established ICD8 to ICD10 alignment and considered ICD8:56,301, ICD8:56,302, ICD8:56,308, ICD8:56,309, and ICD10:K50s as CD diagnosis, and ICD8:56,319, ICD8:56,904, and ICD10:K51s as UC diagnosis [13, 24]. A minimum of two IBD-specific registrations were required to classify individuals with IBDs, including primary (type A) and secondary (type B) diagnoses. The primary diagnosis (type A) indicates the main condition for hospital admission or treatment, while the secondary diagnosis (type B) represents other significant conditions identified during the hospital stay. For each patient, the first recorded IBD diagnosis was used to define the date of diagnosis and the second diagnosis was used to specify the sub-types, i.e., either CD or UC.

The CHB-CID:IBD cohort includes all four mentioned sub-cohorts, consisting of 10,626 patients diagnosed with IBD (Females = 5739 & Males = 4887) with an average age of 55.6 years as of May 2023 (Table 1). In addition, it includes 314,224 individuals without IBD (Females = 163,633 & Males = 150,591) with an average age of 61.9 years (Table 1). All clinical statistics are based on the CHB-CID:IBD cohort with all sub-cohorts except for TARCID for practical reasons. Notably, the next update will include TARCID data.

## Genetic, clinical and laboratory measurements

The CHB-CID:IBD cohort covers genetic and clinical data on all individuals. The participants have been genotyped using Illumina OmniExpress chip [18] or Illumina Infinium Global Screening Array [25] and imputed by deCODE genetics, Reykjavik, Iceland, as described previously, using a whole-genome sequenced reference panel of approx. 50,000 individuals of mixed origin, including 10,800 Danish individuals [26–28]. The CHB-TARCID:IBD cohort is equipped with ≈ 18 million genomic variants, excluding rare variants with minor allele frequency less than 1%.

**Fig. 2:**
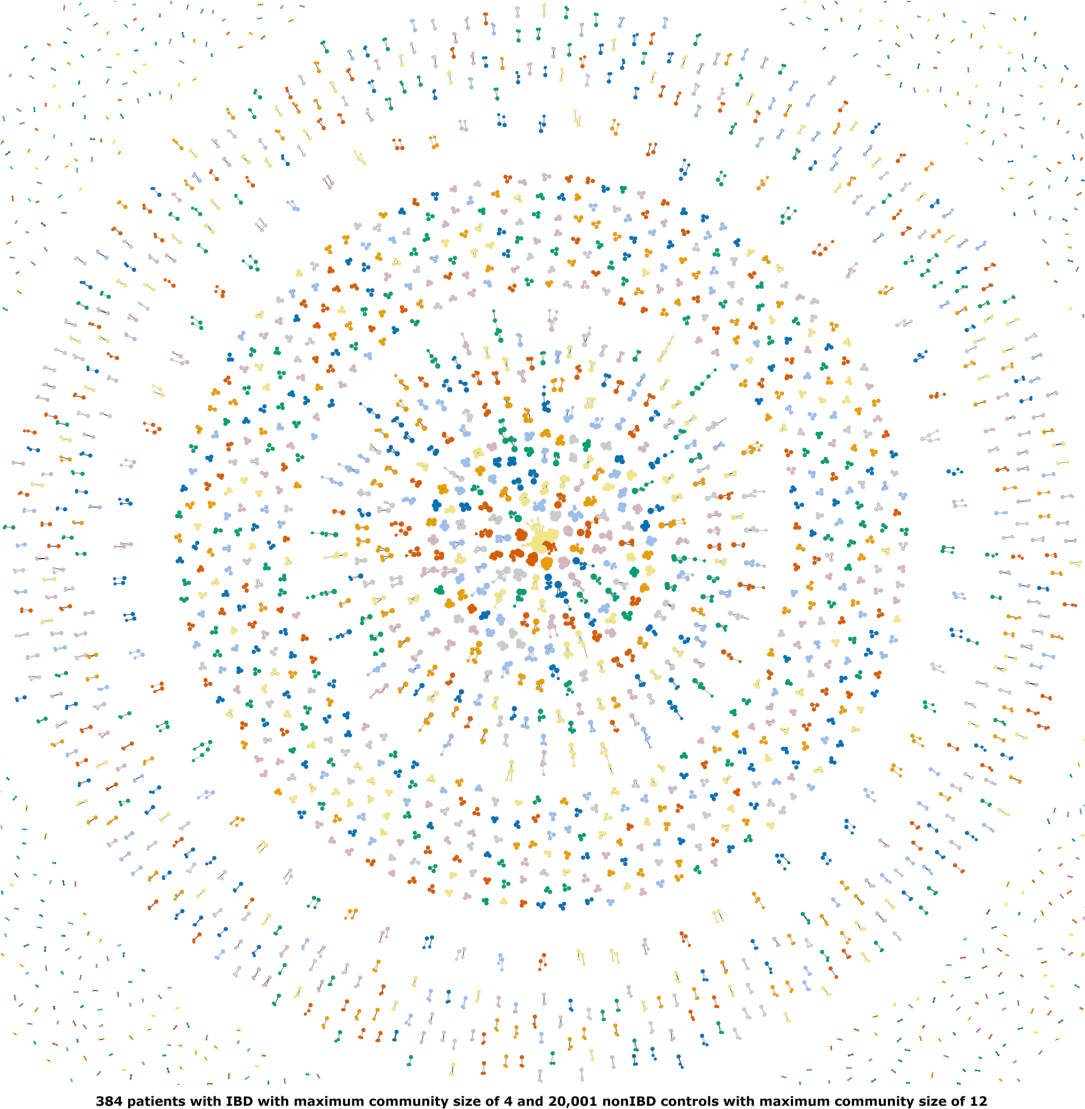
First- and second-degree genetic relationship network among patients with IBD (IBD *vs* IBD) and among non-IBD controls (non-IBD *vs* non-IBD) confirmed the absence of large genetically related communities. Due to the figure size and contrast limitations, a small proportion of the two-member-only communities are shown. Colors are to distinguish the adjacent communities easily. Nodes represent individuals. Node sizes are based on the number of first- and/or second-degree genetic relationships, i.e., the larger the node, the greater the number of genetic relationships

**Fig. 3:**
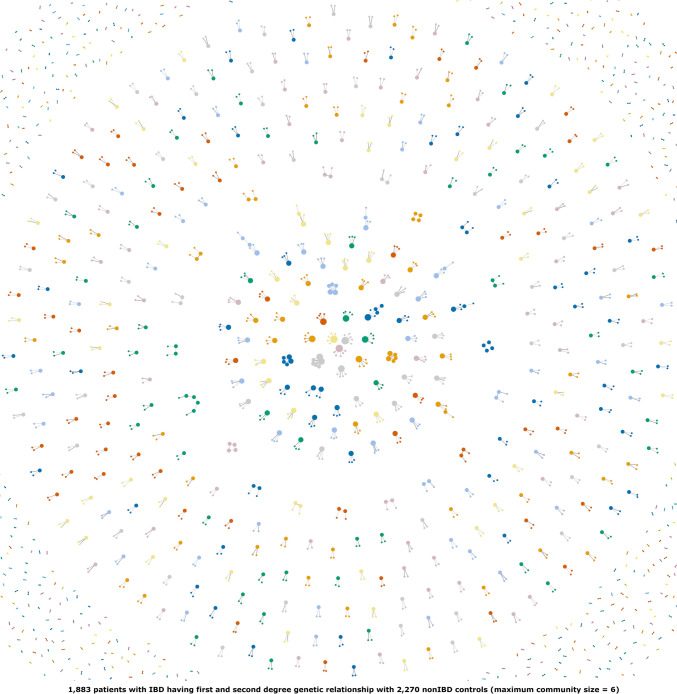
First- and second-degree genetic relationship network between patients with IBD and non-IBD controls, i.e., IBD *vs* non-IBD genetic relationships. Due to the figure size and contrast limitations, a small proportion of the two-member-only communities are shown. Colors are to distinguish the adjacent communities easily. Nodes represent individuals. Node sizes are based on the number of first- and/or second-degree genetic relationships, i.e., the larger the node, the greater the number of genetic relationships

We take several measures to adjust for possible substratification in the cohort, including removing individuals that are outliers in ancestry analysis and adjusting form remaining population substructure by including genetic principle components and co-variates in the association analysis. The examination of genetic relationships [29], found no duplicated samples or monozygotic twins within the cohort, i.e., kinship coefficients were smaller than 0.328. Furthermore, there were not large communities among patients with IBD as well as among non-IBD controls, having first- and second-degree genetic relationships (Fig. 2). First- and second-degree genetic relationships represent genetic similarities at the level of parent vs children, among siblings, grandparent vs grandchildren, among half siblings, aunt/uncle vs niece/nephew, and among double-first cousins. Family-based genome-wide association studies leverage within-family genetic variation to minimize confounding in estimates of direct genetic effects, thereby enhancing power and robustness [30]. Most notably, through the examination of genetic relationships, we found 1,883 patients with IBD having first- and second-degree genetically related non-IBD control counterparts within the CHB-CID:IBD cohort (Fig. 3).

As mentioned earlier, the clinical information is derived from the sub-cohorts, the Danish national health registries[14], and electronic health records in BigTempHealth [15] (Fig. 1a). The CHB-CID:IBD cohort includes information from the following Danish national health registries: the Danish Civil Registration System [14], the National Patient Register [31], and the Danish Laboratory Database [32] (Fig. 1a). The Danish Civil Registration System was initiated in 1968 and assigns a unique ten-digit Civil Personal Register number to all persons, which allows individual-level record linkage and data recovery from different databases [14].

The National Patient Register [31] was established in 1977 and provides clinical data for non-psychiatric inpatients since 1977 and for psychiatric inpatients, and from emergency department and outpatient speciality clinic contacts since 1995 [31]. The National Patient Register contains detailed information on patients, procedures, diagnoses, medication, surgeries, and other healthcare services provided by hospitals [31].

The Danish Laboratory Database [32] includes laboratory assay results from January 2008 and covers most Danish laboratories (3928 different laboratory examinations carried out in 81 laboratories). BigTempHealth covers all electronic patient records in two of the Danish health regions (Fig. 1b), i.e., the Capital Region of Denmark and Region Zealand, from 2006 to 2016 [15]. BigTempHealth [15] is used for text mining, such as extraction of information on smoking.

High-quality clinical information is available for the cohort. In addition to information from the Danish registries, detailed clinical data is retrieved from questionnaires, prospective studies, and patient records. This captures details on disease activity, treatment outcomes, and smoking habits that are not routinely included in registries. Particularly, smoking information will be available for all sub-cohorts (CHB, DBDS, TARCID, and DRB) but not for all participants. The comprehensive diagnosis and laboratory examination records enable the inclusion of diet-related factors such as vitamin and mineral levels in the blood, into the studies.

## Key features and outcomes

**Fig. 4:**
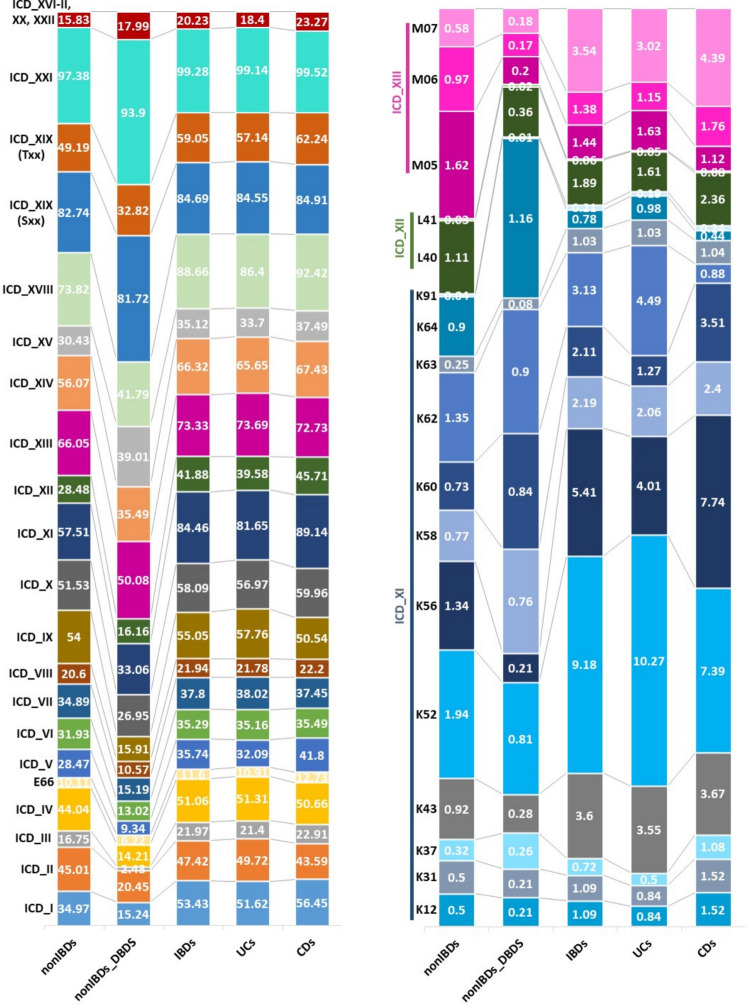
Percentage of individuals with different diagnoses and co-morbidities based on ICD10 classification. ICD10_I: Certain infectious and parasitic diseases, ICD10_II: Neoplasms, ICD10_III: Diseases of the blood and blood-forming organs and certain disorders involving the immune mechanism, ICD10_IV: Endocrine, nutritional and metabolic diseases, E66: Obesity, ICD10_V: Mental and behavioural disorders, ICD10_VI: Diseases of the nervous system, ICD10_VII: Diseases of the eye and adnexa, ICD10_VIII: Diseases of the ear and mastoid process, ICD10_IX: Diseases of the circulatory system, ICD10_X: Diseases of the respiratory system, ICD10_XI: Diseases of the digestive system (IBDs excluded), ICD10_XII: Diseases of the skin and subcutaneous tissue, ICD10_XIII: Diseases of the musculoskeletal system and connective tissue, ICD10_XIV: Diseases of the genitourinary system, ICD10_XV: Pregnancy, childbirth and the puerperium, ICD10_XVI: Certain conditions originating in the perinatal period, ICD10_XVII: Congenital malformations, deformations and chromosomal abnormalities, ICD10_XVIII: Symptoms, signs and abnormal clinical and laboratory findings, not elsewhere classified, ICD10_XIX_S00–S99: Injury, poisoning and certain other consequences of external causes, ICD10_XIX_T00–T98: Injury, poisoning and certain other consequences of external causes, ICD10_XX: External causes of morbidity and mortality, ICD10_XXI: Factors influencing health status and contact with health services, ICD10_XXII: Codes for special purposes, K12: Stomatitis and related lesions, K31: Other diseases of stomach and duodenum, K37: Unspecified appendicitis, K43: Ventral hernia, K52: Other noninfective gastroenteritis and colitis, K56: Paralytic ileus and intestinal obstruction without hernia, K58: Irritable bowel syndrome, K60: Fissure and fistula of anal and rectal regions, K62: Other diseases of anus and rectum, K63: Other diseases of intestine, K64: Haemorrhoids and perianal venous thrombosis, K91: Postprocedural disorders of digestive system, L40: Psoriasis, L41: Parapsoriasis, M05: Seropositive rheumatoid arthritis, M06: Other rheumatoid arthritis, M07: Psoriatic and enteropathic arthropathies, IBD: inflammatory bowel disease, CD: Crohn’s disease, and UC: Ulcerative colitis. UC, DC, and IBDs are based on minimum two type A and/or type B IBD diagnoses

A description of the heterogeneity of patients with IBD is essential for improving disease characterisation and conducting genetic analyses to enhance disease management. Key data summarising this heterogeneity within the cohort is briefly described below, with details provided in Table 1–3 and Fig. 4.

Regarding participant sex and age, the ratio between men and women was nearly 1 among patients with IBD (n = 10,626: Females = 54.0% & Males = 46.0%, Table 1). Table 2 presents the data on patient contacts with the hospital, covering both inpatient and outpatient visits. The average age at first IBD diagnosis was 42.3 years, younger for CD than UC cases (39.8 *vs* 43.9; two-tailed t-test *P*-value = 3.2 × 10^–18^, Table 2). Further, the median number of contacts was 12, with patients with CD having more contacts than patients with UC (15 and 10, respectively). Patients diagnosed during 1986–1995, 1996–2005, 2006–2015, and 2016–2023 show a progressively higher average number of annual contacts than those diagnosed in 1976–1985 (Table 2).

**Table 2:** The IBD hospital contact statistics from inpatient and outpatient records

		CHB-CID:IBD excluding TARCID		
		UCs^a^	CDs^a^	IBDs^a^
Number of individuals		4169	2505	6674
IBD-diagnoses per person (avg.|median)		17.52|10	26.64|15	20.94|12
Avg. age at first IBD diagnosis		43.9	39.8	42.3
Avg. number of IBD diagnosis per year^b^		1.84	2.57	2.11
First IBD diagnosis year 1976–1985	Number of cases	351	178	529
	Avg. number of IBD diagnosis	20.19	41.5	27.36
	Avg. age at first IBD diagnosis	32.1	30.5	31.5
	Avg. number of IBD diagnosis per year^b^	0.50	1.04	0.68
First IBD diagnosis year 1986–1995	Number of cases	533	289	822
	Avg. number of IBD diagnosis	21.74	39.58	28.01
	Avg. age at first IBD diagnosis	37.0	31.1	34.9
	Avg. number of IBD diagnosis per year^b^	0.74	1.30	0.94
First IBD diagnosis year 1996–2005	Number of cases	951	516	1467
	Avg. number of IBD diagnosis	17.74	30.27	22.14
	Avg. age at first IBD diagnosis	43.3	37.2	41.2
	Avg. number of IBD diagnosis per year^b^	0.87	1.45	1.08
First IBD diagnosis year 2006–2015	Number of cases	1451	895	2346
	Avg. number of IBD diagnosis	16.6	23.02	19.03
	Avg. age at first IBD diagnosis	46.4	41.3	44.4
	Avg. number of IBD diagnosis per year^b^	1.66	2.19	1.86
First IBD diagnosis year 2016–2023	Number of cases	883	627	1510
	Avg. number of IBD diagnosis	15.22	18.65	16.64
	Avg. age at first IBD diagnosis	49.1	46.5	48.0
	Avg. number of IBD diagnosis per year^b^	4.36	5.04	4.65

^a^UC, DC, and IBDs are based on minimum two type A and/or type B IBD diagnoses. ^b^Measurements are based on the number of years from first IBD diagnosis to May 2023 for alive individuals and to the date of death for deceased individuals. avg.: average, IBD: inflammatory bowel disease, CD: Crohn’s disease, and UC: Ulcerative colitis

Table 3 describes laboratory examinations, treatment with biologics, and intestinal-related surgery statistics. Faecal Calprotectin and plasma C-reactive protein (CRP) are essential in assessing disease activity in IBD. We noticed higher levels of faecal Calprotectin among IBD cases compared to non-IBD patients and non-IBD blood donors (Average: 507.6 *vs* 158.5 & 107.3 mg/kg; two-tailed t-test *P*-value < 2.4 × 10^–225^, Table 2), and much higher levels when measured within three days of first IBD diagnosis (IBDs: average = 1247.3 mg/kg & median = 1210 mg/kg, UCs: average = 1385.4 mg/kg & median = 1420 mg/kg, CDs: average = 1028.2 mg/kg & median = 768.5 mg/kg). This contrasts with plasma CRP, except for patients with CD within the first three days of IBD diagnoses, who had higher levels of plasma CRP compared to non-IBD controls (Average: 72.1 vs 41.7 mg/L, Median: 45 vs 13 mg/L; two-tailed t-test *P*-value = 6.6 × 10^–62^).

**Table 3:** Genetics, laboratory examinations, treatments, and intestinal related surgery statistics

	CHB-CID:IBD excluding TARCID sub-cohort				
	non-IBD controls	non-IBD controls: DBDS-only	UCs^a^	CDs^a^	IBDs^a^
Number of individuals (Ind.)	309,677	91,871	4169	2505	6674
Ind. not genotyped	0	0	0	0	0
Ind. with no laboratory test	5129	3820	< 10	< 5	10
Number of laboratory tests per person (average|median)	40.4|25	11.5|8	68.1|52	78.5|62	71.9|56
Ind. with CRP^b^ measurement	279,144	66,032	4128	2494	6622
CRP level (mg/L: average|median)^c^	41.7|13	21.2|4	31.6|8	31.6|8	31.6|8
Ind. with no CRP measurement	30,533	25,839	41	11	52
Number of CRP measurements per person (average|median)	22.1|0	3.4|2	44.5|30	55.4|41	48.6|34
Ind. with FCAL^d^ measurement	10,299	3033	2065	1508	3573
FCAL level (mg/kg: average|median)^c^	158.5|31	107.3|30	587.7|164	423.3|138	507.6|150
Ind. with no FCAL measurement	299,378	88,838	2104	997	3101
Number of FCAL measurements per person (average|median)	0.1|0	0.1|0	2.5|0	4|1	3.1|1
Ind. treated with BOHJ18^e^	5598 (1.8%)	224 (0.2%)	886 (21.3%)	846 (33.8%)	1732 (25.9%)
Ind. treated with BOHJ18^e^ after IBD diagnosis	–	–	874 (20.9%)	831 (33.2%)	1705 (25.6%)
Ind. treated with BOHJ19^f^	7885 (2.6%)	336 (0.4%)	324 (7.8%)	227 (9.1%)	551 (6.2%)
Ind. treated with BOHJ19^f^ after IBD diagnosis	–	–	310 (7.5%)	225 (8.9%)	535 (6.0%)
Ind. treated with BOHJ19H4^g^	6 (0.0%)	< 5	205 (4.9%)	173 (6.9%)	378 (5.7%)
Ind. treated with BOHJ19H4^g^ after IBD diagnosis	–	–	205 (4.9%)	173 (6.9%)	378 (5.7%)
Ind. treated with BOHJ28^h^	613 (0.2%)	100 (0.1%)	26 (0.7%)	11 (0.4%)	37 (0.4%)
Ind. treated with BOHJ28^h^ after IBD diagnosis	–	–	25 (0.6%)	10 (0.4%)	35 (0.4%)
Ind. treated with BOHJ28D^i^	64 (0.02%)	< 5	15 (0.4%)	< 5 (< 0.2%)	17 (0.6%)
Ind. treated with BOHJ28D^i^ after IBD diagnosis	–	–	15 (0.4%)	< 5 (< 0.2%)	17 (0.3%)
Ind. treated with BOHJ18^e^, BOHJ19^f^ and/or BOHJ28^h^	13,478 (4.4%)	636 (0.7%)	997 (23.9%)	887 (35.4%)	1884 (28.2%)
Ind. treated with BOHJ18^e^, BOHJ19^f^ and/or BOHJ28^h^ after IBD diagnosis	–	–	978 (23.5%)	870 (34.7%)	1848 (27.7%)
Ind. with KJFA^j^ surgeries	26,069 (8.42%)	3453 (3.76%)	631 (15.14%)	439 (17.52%)	1070 (16.03%)
Ind. with KJFB^k^ surgeries	11,495 (3.71%)	349 (0.38%)	339 (8.13%)	786 (31.38%)	1125 (16.86%)
Ind. with KJFC^l^ surgeries	878 (0.28%)	15 (0.02%)	50 (1.2%)	90 (3.59%)	140 (2.1%)
Ind. with KJFF^m^ surgeries	4261 (1.38%)	93 (0.1%)	374 (8.97%)	288 (11.5%)	662 (9.92%)
Ind. with KJFG^n^ surgeries	2976 (0.96%)	93 (0.1%)	500 (11.99%)	305 (12.18%)	805 (12.06%)
Ind. with KJFH^o^ surgeries	702 (0.23%)	5 (0.01%)	613 (14.7%)	183 (7.31%)	796 (11.93%)
Ind. with KJFK^p^ surgeries	3158 (1.02%)	112 (0.12%)	185 (4.44%)	165 (6.59%)	350 (5.24%)
Ind. with KJFL/M/W^q^ surgeries	428 (0.14%)	28 (0.03%)	22 (0.53%)	15 (0.6%)	37 (0.55%)
Ind. with KJGA^r^ surgeries	3476 (1.12%)	695 (0.76%)	115 (2.76%)	73 (2.91%)	188 (2.82%)
Ind. with KJGB^s^ surgeries	3360 (1.09%)	113 (0.12%)	361 (8.66%)	113 (4.51%)	474 (7.1%)
Ind. with KJGC/D/W^t^ surgeries	571 (0.18%)	25 (0.03%)	28 (0.67%)	25 (1%)	53 (0.79%)
Ind. with KJHA–C^u^ surgeries	9314 (3.01%)	2068 (2.25%)	347 (8.32%)	351 (14.01%)	698 (10.46%)
Ind. with KJWA–W^v^ surgeries	3938 (1.27%)	171 (0.19%)	193 (4.63%)	187 (7.47%)	380 (5.69%)
Ind. with one or more of the above-mentioned surgeries	48,525 (15.67%)	6093 (6.63%)	1665 (39.94%)	1352 (53.97%)	3017 (45.21%)

^a^UC, DC, and IBDs are based on minimum two type A and/or type B IBD diagnoses. ^b^CRP: C-reactive protein. ^c^Based on individuals with the corresponding lab test (individuals with no lab test are not included in the analysis). ^d^FCAL: Faecal Calprotectin. ^e^BOHJ18: Biological drugs including TNFi (infliximab, etanercept, adalimumab, golimumab, and certolizumab pegol), interleukin inhibitors (anakinra, tocilizumab, ustekinumab, canakinumab, secukinumab, brodalumab, guselkumab, dupilumab, and sarilumab). ^f^BOHJ19: including antibody treatment: anti-IgE antibody (omalizumab), anti-vascular endothelial growth factor(bevacizumab and ramucirumab), anti-epidermal growth factor receptor (zalutumumab and panitumumab), anti-CTLA4 monoclonal antibody (ipilimumab and tremelimumab), anti-CD30 monoclonal antibody (brentuximab vedotin and mogamulizumab), anti-IGF-1 antibody (dalotuzumab), other monoclonal antibodies (rilotumumab, nivolumab, pertuzumab, vedolizumab, obinutuzumab, belimumab, durvalumab, daratumumab, and elotuzumab), anti-IL5 monoclonal antibody (reslizumab, mepolizumab, and benralizumab), anti-PD-L1 antibody (avelumab, atezolizumab, pembrolizumab, and cemiplimab), bi-specific T-cell antibody (blinatumomab), anti-PDGFR antibody (olaratumab), anti-CGRPR ntibody (erenumab and fremanezumab), anti-IL23 antibody (risankizumab), anti-CD25 monoclonal antibody (basiliximab). ^g^BOHJ19H4: Vedolizumab. ^h^BOHJ28: Other immunomodulating treatments (teriflunomid, dimethylfumarat, glatirameracetat, tofacitinib). ^i^BOHJ28D: Tofacitinib. ^j^KJFA: local surgeries on intestine. ^k^KJFB: bowel resections. ^l^KJFC: intestinal anastomoses without resections. ^m^KJFF: ostomy. ^n^KJFG: operations on enterostomy and intestinal reservoir. ^o^KJFH: total colectomies. ^p^KJFK: adhesiolysis of intestinal obstruction. ^q^KJFL/M/W: operation for intestinal obstruction without adhesiolysis/operative bowel lavage/other operations on small and large intestine. ^r^KJGA: local operations on the rectum, ^s^KJGB: Rectal resections. ^t^KJGC/D/W: rectal reconstructions/operations on the perirectal tissue/other operations on the rectum. ^u^KJHA–C: biopsies, incisions and excisions of pathological tissue in anal canal and perianal tissue/operations for hemorrhoids and anal mucosal prolapse/anorectal reconstructions. ^v^KJWA–W: reoperations after surgery on the gastrointestinal tract. IBD: inflammatory bowel disease, CD: Crohn’s disease, UC: Ulcerative colitis, and Ind.: Individuals

Traditionally, TNFi has been the first-line biologic therapy prescribed for patients with severe IBD in Denmark and it has been generally available since the 2000s. In total, 25.6% of patients with IBD (20.9% UC, 33.2% CD) were treated with biologics after IBD diagnosis (BOHJ18, Table 3). In total, 45.2% of patients with IBD had intestinal-related surgeries; patients with CD had a high occurrence of bowel resections and patients with UC had a high occurrence of total colectomies (Table 3). Co-morbidities are important manifestations of diseases adding to the phenotypic diversity (Fig. 4). A higher proportion of patients with IBD registered psoriatic and enteropathic arthropathies (ICD10:MO7) as well as intestinal diseases (ICD10_XI excluding IBDs) compared to non-IBD controls (Fig. 4).

The information mentioned will be further explored in planned genetic analyses. Furthermore, the CHB-CID:IBD sub-cohort results have previously been published as CHB [25], TARCID [22, 23], and DBDS [17, 18]. The CHB, DBDS and TARCID sub-cohorts have been used to study genetics of various conditions, such as rheumatoid arthritis, cardiovascular diseases, essential tremor, iron homeostasis and drug treatment outcomes [33–38]. For example, our study identified new non-HLA loci to be associated with rheumatoid arthritis. Finally, genetic variants associated with treatment response were identified in IBD, psoriasis, and rheumatoid arthritis.

## Strengths and weaknesses

The CHB-CID:IBD cohort has several strengths. The Danish registries offer complete coverage of the entire country's population. In addition, the validity of the IBD diagnosis has been found to be very high with a positive predictive value of 95% [39]. Therefore, this study provides an extraordinary opportunity to conduct populations-based studies on IBDs by providing genetic and clinical information from an extensive cohort. Moreover, results from CHB-CID:IBD can be replicated using Danish cohorts such as BELIEVE and the Danish IBD Biobank as well as international cohorts of deCODE [40], FinnGen [41], UKBiobank [42], and IIBDGC [7]. BELIEVE includes patients with chronic inflammatory disease who were prescribed TNFi treatment between June 2017 and March 2019 [43]. BELIEVE includes more than 100 patients with IBD. The Danish IBD Biobank project was initiated in May 2019 and includes 840 patients with IBD [44]. In addition, the CHB-CID:IBD cohorts' data can be enriched by adding more data types, such as omics data from plasma samples of the DBDS subcohort. Finally, CHB-CID:IBD results can be further explored by downstream molecular and omics analyses through genotype recall studies in the DBDS sub-cohort. The main weakness of the CHB-CID:IBD cohort is the lack of information on patient-related outcomes and other lifestyle measures such as diet. In conclusion, the CHB-CID:IBD cohort provides a unique platform to study the role of genetics and gene-environment interactions in IBD. Integrating genetic data with clinical data will allow us to stratify patients according to disease characteristics and pinpoint patient heterogeneity. We are confident that the models based on results from CHB-CID:IBD will establish a foundation for more individualised treatment, benefiting patients with IBD and society as a whole.

## Data access

The CHB-CID:IBD cohort can be accessed for research through collaboration. All collaboration enquiries should be submitted to the steering committee and principal investigators Vibeke Andersen (TARCID sub-cohort; va@rsyd.dk), Bente Glintborg (DRB sub-cohort; bente.glintborg.01@regionh.dk), Sisse Rye Ostrowski and Ole Birger Vesterager Pedersen (CHB & DBDS sub-cohorts; Sisse.Rye.Ostrowski@regionh.dk and olbp@regionsjaelland.dk).

## Abbreviations

CD: Crohn’s disease
CHB-CID: Copenhagen Hospital Biobank-chronic inflammatory disease
CRP: C-reactive protein
DBDS: Danish blood donor study
DRB: The Danish rheumatologic biobank
FCAL: Faecal calprotectin
ICD: International statistical classification of diseases and related health problems revision
IBD: Inflammatory bowel disease
TARCID: Targeted treatment of chronic inflammatory disease
TNFi: Tumor necrosis factor inhibitors
UC: Ulcerative colitis

## Declarations

### Concent for publication

ChatGPT (https://chat.openai.com/) assisted copy editing was used and authors reviewed and revised the final version, taking full responsibility for the content of the publication.
